# Effect of traditional Chinese exercise on the quality of life and depression for chronic diseases: a meta-analysis of randomised trials

**DOI:** 10.1038/srep15913

**Published:** 2015-11-03

**Authors:** Xueqiang Wang, Yanling Pi, Binglin Chen, Peijie Chen, Yu Liu, Ru Wang, Xin Li, Yi Zhu, Yujie Yang, Zhanbin Niu

**Affiliations:** 1 Department of Sport Rehabilitation, Shanghai University of Sport, Shanghai, China; 2 Department of Rehabilitation Medicine, Shanghai Shangti Orthopaedic Hospital, Shanghai, China; 3 Department of Rehabilitation Medicine, Shanghai Punan Hospital, Shanghai, China; 4 Key Laboratory of Exercise and Health Sciences of Ministry of Education, Shanghai University of Sport, Shanghai, China; 5 Rehabilitation therapy center, Hainan Province Nongken General Hospital, Haikou, 15 China; 6 Second School of Clinical Medical, Nanjing University of Chinese Medicine, Nanjing, China

## Abstract

Traditional Chinese exercise (TCE) has many uses in the prevention and treatment of chronic diseases. However, there is no consensus regarding the benefit of TCE for chronic diseases. Our objective is to examine the effect of TCE on the quality of life and depression for chronic diseases by performing a meta-analysis of randomized controlled trials (RCTs). We only cover published RCTs. The outcome measures included quality of life and depression. Sixty articles with a total of 4311 patients were included. The pooling revealed that TCE could improve the SF-36 physical function subscale in the short term [SMD (95% CI) = 0.35 (0.13, 0.56), P = 0.002] and mid-term [SMD (95% CI) = 0.49 (0.12, 0.85), P = 0.009], GHQ [SMD (95% CI) = −0.68 (−1.26, −0.09), P = 0.02], the Center for Epidemiologic Studies depression scale in the short term [SMD (95% CI) = −0.86 (−1.42, −0.31), P = 0.002] and mid-term [SMD (95% CI) = −0.41 (−0.64, −0.18), P < 0.001]. The meta-analysis of RCT demonstrates that TCE can significantly improve the quality of life and depression of patients with chronic diseases. These findings provide useful information for patients with chronic diseases as well as for medical staff.

Chronic diseases are the leading cause of death in developed and developing countries^1^. These long-term diseases drastically affect the quality of life of afflicted patients and can cause depression of afflicted patients. Indeed, health-related quality of life (physical, psychological status) is increasingly important in people suffering from chronic diseases. According to the World Health Organization^2^, more than 36 million people in the world are killed by chronic diseases each year, and approximately 80% of these deaths, accounting for 29 million people, are from low- and middle-income countries. The five main types of chronic diseases include cardiovascular and cerebrovascular diseases, chronic respiratory diseases, diabetes, cancers and musculoskeletal disorders^2^,^3^,^4^. Given the prevalence of chronic diseases and mental illnesses, the World Economic Forum concluded that the world would sustain a cumulative output loss of $47 trillion between 2011 and 2030, of which nearly $30 trillion would be attributable to cardiovascular diseases, chronic pulmonary diseases, diabetes, and cancers^4^. Therefore, low-cost, easily accessible, and side effect-free programs must be developed to cure such chronic diseases.

Exercise is generally well accepted as significantly contributing to the prevention and treatment of chronic diseases^5^. Traditional Chinese exercise (TCE) is a representative form of exercise that is becoming increasingly popular worldwide for the improvement of health and well-being. TCE such as Tai Chi, Qigong, and Baduanjin does not require the use of equipment, is low in cost and easy to learn^6^,^7^,^8^. TCE has been used for 2000 years and is also a promising^9^, low-risk intervention that can help improve quality of life and alleviate depression in patients with chronic diseases^10^,^11^,^12^.

TCE includes different types of exercise; the main types are Tai Chi, Qigong, Baduanjin, and Liuzijue, among others. Tai Chi, which is also called “taiji,” “taijichuan” or “taijiquan,” is a famous form of TCE worldwide. Tai Chi is a type of traditional mind body exercise, that developed as a martial art and a means of self-defense in China. Qigong is a general form of TCE and comprises exercises for postural control, coordinated breathing and meditation. *Qi* refers to vital energy, and *gong* means discipline. Baduanjin translates to the Eight Section Brocades, which refers to eight individual movements for improving general health. Liuzijue is a form of breathing exercise in China that was passed down from ancient times^6^,^7^,^8^,^9^,^10^,^11^,^12^,^13^. The practice of TCE usually focuses on a combination of physical and mental exercises.

Most TCE is not only exercise therapy, but also includes meditation field. Because of the meditative aspect, TCE could also improve psychological well-being and reduce stress. According to Chang *et al.*^14^, TCE (e.g., Tai Chi) is theorized to improve cognition by enhancing brain activation through meditation. In addition, Wayne *et al.*^15^ had proposed a relationship between Tai Chi and social interaction, and this positive linkage has even extended to brain function. Based upon the model of Chang *et al.*^14^, TCE could bring positive efficacy in cognition via multiple pathways, including motor function, cardiovascular function, coordination function, social interaction, and meditation.Although TCE is widely performed to prevent and treat chronic diseases, studies on TCE have not reached a consensus with regard to how such exercise can improve the quality of life and alleviate depression in patients with chronic diseases^10^,^11^,^12^,^16^,^17^. Similarly, we have yet to find any meta-analysis that has assessed the effect of TCE on the quality of life and depression of patients with chronic diseases. Previous systematic reviews have focused on one type of TCE (such as Tai Chi) for chronic diseases. Therefore, this current meta-analysis aims to identify the effects of TCE on the quality of life and depression of patients with chronic diseases. Additionally, the meta-analysis provides an overall assessment of the effect of TCE on the quality of life and mental health of patients with chronic diseases as well as of the different TCE methods used to treat people with chronic diseases.

## Methods

### Protocol and registration

The meta-analysis was performed and reported in accordance with the Preferred Reporting Items for Systematic Reviews and Meta Analyses (PRISMA) guidelines. The protocol was registered prior to conducting the review. Systematic review registration: http://www.crd.york.ac.uk/PROSPERO. PROSPERO registration number: CRD42013006474.

### Search strategy

We searched for relevant studies that were published between January 1957 and January 2015 from several electronic data sources, including PubMed, EMBASE, Web of Science, the Cochrane Library, EBSCO (CINAHL), and China National Knowledge Infrastructure. No language restrictions were employed. The search was limited to randomized controlled trials (RCTs). All of the electronic search strategies for all databases are provided in Supplementary Table S1.

### Inclusion criteria

1. Types of studies: We only covered published articles with completed RCTs.
2. Types of participants: We included articles wherein the participants suffered from five main clusters of chronic diseases: cardiovascular and cerebrovascular diseases (e.g., stroke and heart attacks), musculoskeletal disorders (e.g., fibromyalgia), chronic respiratory diseases (i.e., chronic obstructed pulmonary disease), cancers, and diabetes.
3. Types of interventions: We only considered articles that compared an intervention group, in which the members performed TCE (e.g., Tai Chi, Qigong, and Baduanjin), with a control group, in which the members performed another intervention (i.e., strength exercise or drug) or that did not undergo any intervention.
4. Types of outcome measures: The outcome measures were quality of life and depression. Outcomes were recorded for three time periods: short term (less than 3 months), mid term (from 3 months to 12 months) and long term (1 year or more).

### Selection of studies

Two authors independently used the same selection criteria to screen the titles, abstracts, and bodies of the relevant articles. The studies that failed to meet the inclusion criteria were removed from the sample. In the case of disagreement, the two authors would discuss or consult a third author.

### Data extraction and management

The following data were extracted from the selected articles: study characteristics (e.g., author and year), participant characteristics (e.g., age and number of subjects), intervention description, trial period duration, assessed outcomes, and time points. The two authors who selected the studies also extracted the data from the included articles. Any disagreement was resolved through discussion, and a third author was consulted in cases where disagreement persisted.

### Quality assessment

We used the PEDro scale^18^ to evaluate the risk of bias for inclusion in the meta-analysis. Using a pre-determined 10-item scale, two review authors independently assessed the methodological quality of each study. The following information was evaluated: random allocation, concealed allocation, baseline comparability, blind subjects, blind therapists, blind assessors, adequate follow-up, intention-to-treat analysis, between-group comparisons, point estimates, and variability. The review authors did not evaluate their own studies. A third author was consulted when a disagreement occurred.

### Statistical analysis

Review Manager software (RevMan5.3) was used to conduct the meta-analysis. The chi-square test and I^2^ statistic were used to evaluate the heterogeneity among the studies. Using a random effects model, the outcome measures from the individual studies were combined through a meta-analysis. If continuous data were reported as the median and within an interquartile range (IQR), the median would be assumed to be equivalent to the mean, and the relationship of the IQR with the standard deviation was roughly SD = IQR/1.35^19^. Given that all variables in the included studies were expressed as continuous data, we used the standardized mean difference or the mean difference and the 95% confidence interval (CI) to analyze the studies. We considered p < 0.05 as statistically significant. Sensitivity analysis was performed by removing each study individually to assess the consistency and quality of the results. Funnel plot asymmetry was employed to assess possible publication bias by Egger’s regression test.

## Results

### Descriptive results

**Figure 1:**
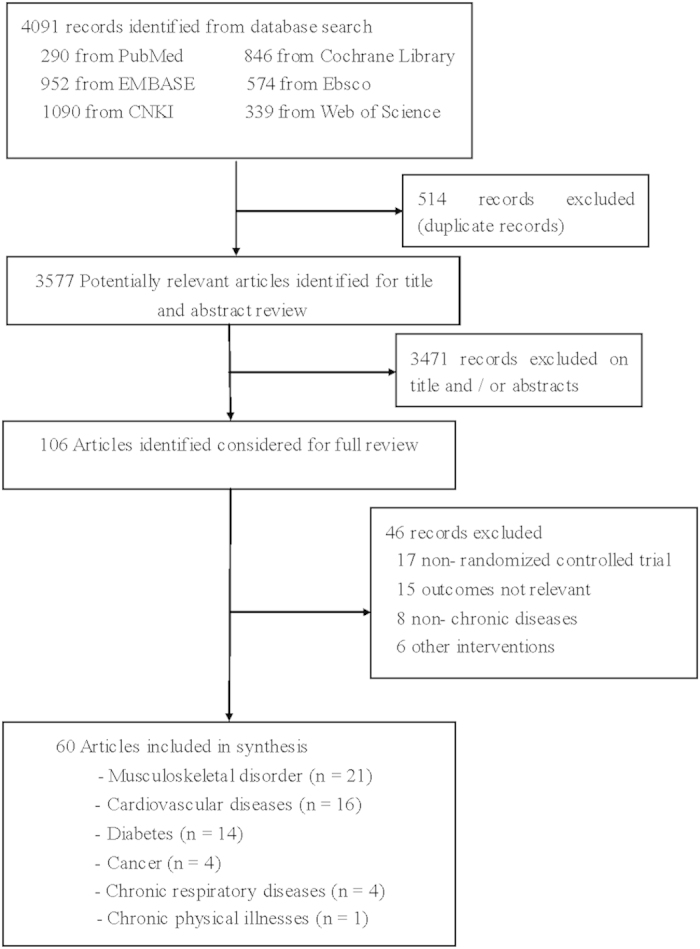
Flow chart of the study selection procedure.

The flow chart of the study selection procedure is outlined in Fig. 1. Of the 106 potentially relevant studies that were identified, 46 were excluded for not completing an RCT or for producing irrelevant outcomes. Thus, we included 4311 patients with chronic diseases from the 60 remaining articles^20^,^21^,^22^,^23^,^24^,^25^,^26^,^27^,^28^,^29^,^30^,^31^,^32^,^33^,^34^,^35^,^36^,^37^,^38^,^39^,^40^,^41^,^42^,^43^,^44^,^45^,^46^,^47^,^48^,^49^,^50^,^51^,^52^,^53^,^54^,^55^,^56^,^57^,^58^,^59^,^60^,^61^,^62^,^63^,^64^,^65^,^66^,^67^,^68^,^69^,^70^,^71^,^72^,^73^,^74^,^75^,^76^,^77^,^78^,^79^ (21 articles focused on musculoskeletal disorders, 16 articles focused on cardiovascular diseases, 14 articles focused on diabetes, 4 articles focused on cancers, 4 articles focused on chronic respiratory diseases, and 1 article focused on chronic physical illnesses). These articles were mainly published in China (n = 21, 35%), USA (n = 15, 25%), Australia (n = 4, 6.67%), Sweden (n = 3, 5%), Korea (n = 3, 5%), Hong Kong (n = 3, 5%), the UK (n = 2, 3.33%), Canada (n = 2, 3.33%), Germany (n = 2, 3.33%), New Zealand (n = 1, 1.67%), Israel (n = 1, 1.67%), and Japan (n = 1, 1.67%). The characteristics of each included study are summarized in Table 1.

### Methodological quality

The methodological quality of all included articles was assessed (Table 2). The generation of the allocation sequence was reported in all articles (n = 60, 100%). A total of 15 articles (25%) conducted allocation concealment. A total of 23 articles (38.33%) blinded the outcome assessors to the treatment allocation. A total of 28 articles (46.67%) had an adequate follow-up period. In addition, 13 articles (21.67%) used the intention to treat as their primary analysis method.

### Quality of life

#### Short-form (SF-36) survey

**Figure 2:**
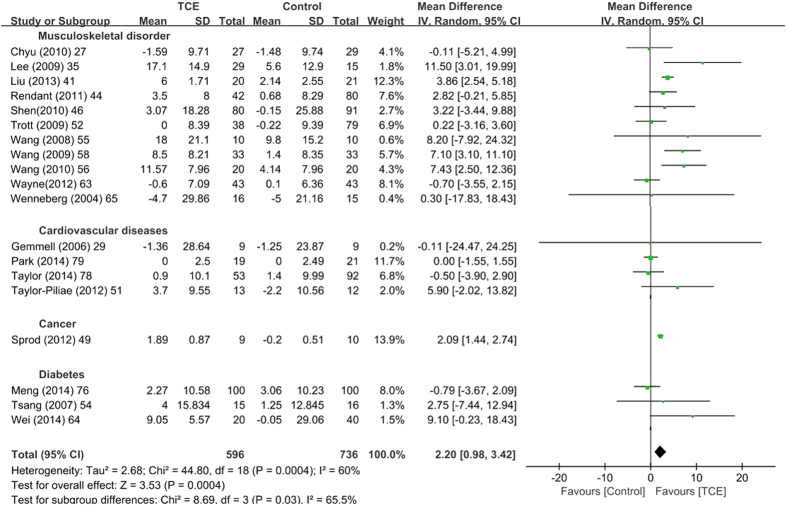
Meta-analyses of traditional Chinese exercises on short form-36 physical function at the short term. SD = standard deviation; 95% CI = 95% confidence intervals; IV = inverse variance.

**Figure 3:**
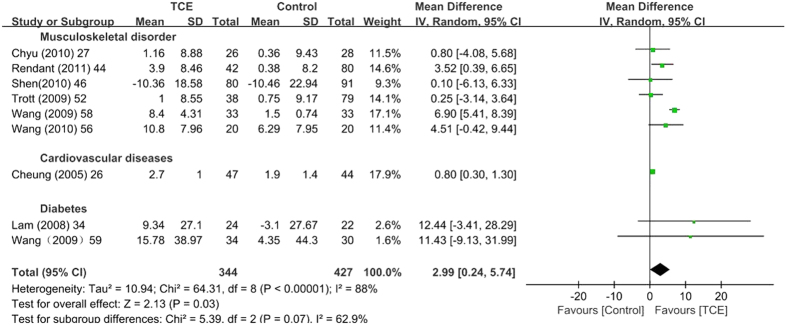
Meta-analyses of traditional Chinese exercises on short form-36 physical function at the mid-term. SD = standard deviation; 95% CI = 95% confidence intervals; IV = inverse variance.

Using a random effects model, the meta-analysis of six studies^35^,^39^,^49^,^62^,^67^,^76^ with 591 patients showed that TCE could improve the total SF-36 score in the short term [SMD (95% CI) = 0.59 (0.32, 0.87), P < 0.001] (Table 3) and mid term [SMD (95% CI) = 0.61 (0.16, 1.05), P = 0.008] (Table 3). The meta-analysis of 22 studies with 1533 patients^26^,^27^,^29^,^34^,^35^,^41^,^44^,^46^,^49^,^51^,^52^,^54^,^55^,^56^,^58^,^59^,^63^,^64^,^65^,^76^,^78^,^79^ that were suitable for inclusion showed that TCE had a significant positive effect on the SF-36 physical function subscale in the short term [SMD (95% CI) = 0.35 (0.13, 0.56), P = 0.002] (Table 3 and Fig. 2) and mid term [SMD (95% CI) = 0.49 (0.12, 0.85), P = 0.009] (Table 3 and Fig. 3). A total of 22 studies with 1502 patients^20^,^27^,^29^,^34^,^35^,^42^,^44^,^46^,^49^,^51^,^52^,^54^,^55^,^56^,^58^,^59^,^63^,^64^,^65^,^76^,^78^,^79^ were included to estimate the effect of TCE on the SF-36 mental health subscale. The TCE group outperformed the control group in terms of the SF-36 mental health subscale in the short term ([SMD (95% CI) = 0.28 (0.11, 0.46), P = 0.002) (Table 3, Supplementary Figure S1) and mid term [SMD (95% CI) = 0.39 (0.08, 0.71), P = 0.02] (Table 3, Supplementary Figure S2). No significant difference was observed among the 15 studies with 935 patients^20^,^26^,^27^,^29^,^34^,^41^,^46^,^49^,^54^,^55^,^59^,^63^,^65^,^76^,^79^ that investigated the SF-36 general health subscale in the short term ([SMD (95% CI) = 0.15 (−0.00, 0.31), P = 0.06] (Table 3, Supplementary Figure S3) and mid term [SMD (95% CI) = 0.05 (−0.24, 0.34), P = 0.73] (Table 3, Supplementary Figure S4). A sensitivity analysis was performed for the total SF-36 score, the SF-36 physical function subscale, the SF-36 mental health subscale, the SF-36 general health subscale, the significance of the results was not changed when studies were removed one by one.

#### General Health Questionnaire (GHQ)

Two studies^22^,^57^ were included to estimate the effect of TCE on the GHQ. The TCE group outperformed the control group in terms of improving the GHQ in a random effects model [SMD (95% CI) = −0.68 (−1.26, −0.09), P = 0.02] (Table 3).

#### WHO quality of life (WHOQOL)

The meta-analysis of four studies^23^,^31^,^53^,^60^ with 287 patients that were suitable for inclusion revealed that TCE had a significant positive effect on the WHOQOL general health subscale [SMD (95% CI) = 0.68 (0.04, 0.47), P = 0.04]. However, TCE did not have a significant effect on the WHOQOL physical health subscale [SMD (95% CI) = 0.13 (−0.59, 0.85), P = 0.73] or the WHOQOL psychological health subscale [SMD (95% CI) = 0.22 (−0.04, 0.47), P = 0.09] (Table 3). The results were affected by one study^60^ for WHOQOL general health, one study^53^ for WHOQOL physical health, and one study^53^ for WHOQOL psychological health in the sensitivity analysis. Therefore, the meta analysis provided weak evidence of the effects of TCE on the WHOQOL.

### Depression

#### Center for Epidemiologic Studies Depression Scale (CES-D)

**Figure 4:**
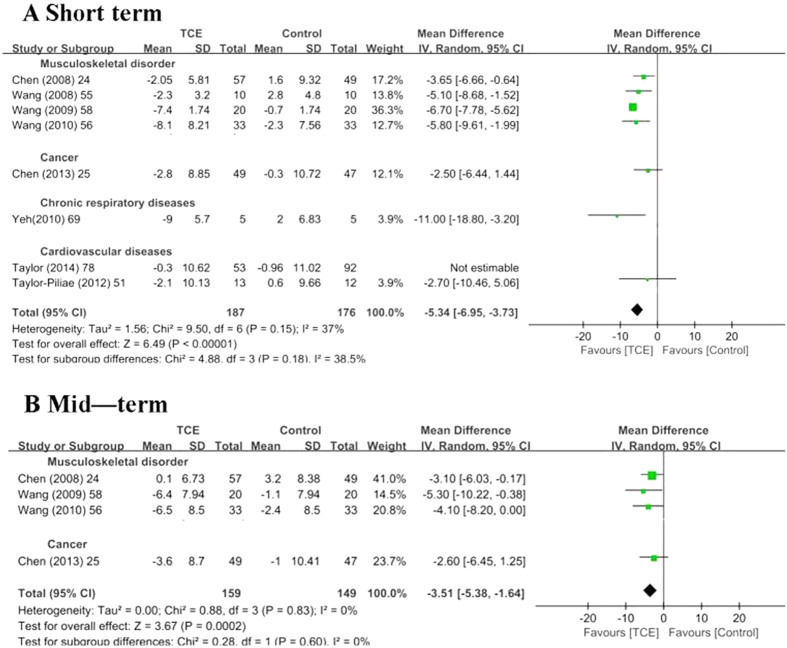
Meta-analyses of traditional Chinese exercises on Center for Epidemiologic Studies Depression Scale. A: in the short term. B: in the mid-term. SD = standard deviation; 95% CI = 95% confidence intervals; IV = inverse variance.

Eight studies^24^,^25^,^51^,^55^,^56^,^58^,^69^,^78^ with 508 patients were included to estimate the effect of TCE on the CES-D. TCE could improve the CES-D in the short term [SMD (95% CI) = −0.86 (−1.42, −0.31), P = 0.002] (Table 3 and Fig. 4A) and mid term [SMD (95% CI) = −0.41 (−0.64, −0.18), P < 0.001] (Table 3 and Fig. 4B). Sensitivity analysis revealed that the pooled result was stable when studies were removed one by one.

#### Self-rating depression scale (SDS)

The meta-analysis of five studies^30^,^33^,^40^,^72^,^75^ with 315 patients that were suitable for inclusion found that TCE had a significant effect on the SDS in the short term [SMD (95% CI) = −0.6 (−0.83, −0.36), P < 0.001] (Table 3, Supplementary Figure S5 A). Sensitivity analysis found that the pooled result was not influenced by individual trials.

#### Beck Depression Inventory (BDI)

Three studies^26^,^31^,^43^ with data from 180 patients were included to assess the effect of TCE on the BDI. TCE had a non-significant positive effect on the BDI in a random effects model [SMD (95% CI) = −0.15 (−0.75, 0.44), P = 0.61] (Table 3). The significance of the result was changed in the sensitivity analysis when one study^26^ was removed, this result offered inferior evidence for the effect of TCE on BDI.

#### Profile of Mood States-Depression (POMS-D)

Three studies^68^,^70^,^71^ with data from 156 patients were used to estimate the effect of TCE on the POMS-D. TCE could improve the POMS-D in the short term (SMD (95% CI) = −1.64 (−2.55, −0.73), P < 0.001) (Table 3, Supplementary Figure S5 B). Sensitivity analysis indicated that the pooled result was not influenced by individual trials.

#### Hamilton Depression Scale (HAMD)

The meta-analysis of three studies^37^,^61^,^73^ with 189 patients that were suitable for inclusion indicated that TCE had a significant effect on the HAMD in the short term [SMD (95% CI) = −1.36 (−1.97, −0.75), P < 0.001] (Table 3, Supplementary Figure S5 C). Sensitivity analysis revealed that the pooled result was stable when studies were removed one by one.

#### Symptom Checklist-90 (SCL-90)

Four studies^21^,^38^,^74^,^77^ presenting data from 284 patients were included to assess the effect of TCE on the SCL-90. The result showed that TCE improved the SCL-90 [SMD (95% CI) = −0.7 (−1.32, −0.08), P = 0.03] (Table 3). Sensitivity analysis indicated that the pooled result was not influenced by individual trials.

### Publication bias

The results of the Egger’s regression test did not reveal any publication bias for the total SF-36 (asymmetry test P = 0.128), SF-36 physical function (asymmetry test P = 0.207), the SF-36 mental health subscale (asymmetry test P = 0.678), the SF-36 general health subscale (asymmetry test P = 0.906), and the CES-D (asymmetry test P = 0.361).

## Discussion

### Summary of findings

Several types of TCE are used to prevent and treat chronic diseases. However, the extant systematic reviews primarily focus on either one type of disease (e.g., cardiovascular disease) or one type of TCE (e.g., Tai Chi). In this review and meta-analysis, we combined all of the evidence from the numerous relevant studies evaluating the various forms of TCE into one review to assess the overall effect of TCE on patients with chronic diseases.

We gathered information on 4311 subjects from 60 articles that provided evidence on the effects of TCE on improving the quality of life and alleviating depression in patients with chronic diseases. The meta-analysis revealed that TCE had a significant positive effect on the quality of life (SF-36 physical function, SF-36 mental health, SF-36 total, GHQ, and WHOQOL general health) and depression (CES-D, SDS, POMS-D, HMAD, SCL-90) in patients with chronic diseases. Therefore, TCE had a significant clinical effect on improving the quality of life and reducing depression in patients with chronic diseases. We used the chi-square test and I^2^ statistic to assess the heterogeneity among the studies, and identified obvious heterogeneity for some outcomes. To solve this problem, sensitivity analysis was conducted for to assess the consistency and quality of the results. Sensitivity analysis revealed that most of the pooled results (SF-36, CES-D, SDS, POMS-D, HAMD, SCL-90) were stable when studies were removed one by one. But the significance of the result (WHOQOL, BDI) was changed through sensitivity analysis, these results offered inferior evidence for the effect of TCE on WHOQOL and BDI.Because TCE does not require the use of equipment, the exercises are low in cost and easy to learn. Chronic patients who performed TCE demonstrated considerable improvements in their conditions^80^,^81^. According to the theory of Traditional Chinese medicine, TCE could help one’s body to dredge the meridians and collaterals, facilitate blood circulation, relax the mind, balance emotion, and regulate the internal organs to enhance one’s physical health and quality of life and to improve one’s psychological state^6^. TCE generally combines postural control, breath regulation and mediation. The primary benefit of TCE stems from the holistic nature, and TCE benefits both physical and psychosocial health. However, the exact mechanism by which TCE affects patients with chronic diseases is complex and remains unclear. Thus, further evidence on the mechanism by which TCE affect chronic diseases should be obtained.

### Strengths and limitations

Relevant articles were searched from a wide range of electronic databases (e.g., PubMed, EMBASE, Web of Science, and Cochrane Library). Considering that TCE originated in China, we searched for relevant information from the largest Chinese information database. The current study was the first meta-analysis to estimate the effects of TCE on the quality of life and depression of patients with chronic diseases by comparing an intervention group with a no intervention group. Given that the selected articles were published in America, Asia, Europe, and Oceania, the results of this study may be generalizable to most parts of the world. Furthermore, most of the included articles were published over the last five years (from 2010 to 2015). To reduce bias and transcription errors, two authors independently performed the study selection, data extraction, and quality assessment processes.

Nevertheless, our meta-analysis had several limitations. First, although all of the included articles were RCTs, only 15 studies (25%) reported how the patient allocation was concealed. According to the intention-to-treat principle, 13 articles (21.67%) used the intention to treat as their primary analysis method. A total of 23 articles (38.33%) blinded the outcome assessors to the treatment allocation. Second, the outcome assessors could not be blinded for the comparison between the TCE group and the no-intervention group, therefore generating potential performance and response biass. Third, most articles had no long-term follow-up period (over one year). Therefore, we did not conduct a meta-analysis to estimate the long-term effect of TCE on chronic diseases. Fourth, we used Egger’s regression test to assess publication bias. Additionally, we systematically searched several electronic databases for publications. Although we found no publication bias, we did not search for any unpublished trials. Fifth, due to the lack of studies with training time ranging from 6 months to 12 months, especially 12 months, we did not perform a subgroup meta analysis focusing on exercise training time. Sixth, some studies included in our meta analysis had small sample sizes. Future meta-analyses including more large-scale, high-quality RCTs are required to obtain further proof of the effects of TCE. Seventh, most of the Chinese studies from our meta analysis were not registered in the international clinical trials registry platform of the World Health Organization. To reduce bias, all studies should be performed in accordance with the standards of clinical trials (e.g., the Consolidated Standards of Reporting Trials statement).

### Implications for policy and practice

Traditional medicine/exercise, including TCE is an important and often underestimated part of health services worldwide^82^. TCE is extensively performed in most countries; however, in many countries and regions, the public, medical professionals, and healthcare policymakers remain confused about the effectiveness, safety, and quality of TCE. In our meta-analysis, we identified both the scientific and clinical importance of TCE. Unlike other exercises, TCE may contribute to improving the quality of life (e.g., SF-36 and GHQ) and reducing depression (e.g., CESD, SDS, BDI) in patients with chronic diseases. These findings provide useful information for chronic disease patients, medical professionals, and healthcare policymakers. As public health professionals, we believe that healthcare policymakers and medical professionals must consider how TCE improves the health of patients with chronic diseases.

### Implications and future research

Further evidence from larger and better quality studies must be collected to determine the effects of TCE on chronic diseases. Most of the articles with small sample sizes that were included in our meta-analysis only observed patients over a short-term follow-up period. Multicenter RCTs with large sample sizes must be conducted to validate the effects of TCE in patients with chronic diseases. Future studies should improve their methodological standards in the following aspects: random allocation, allocation concealment, long-term follow-up, intention-to-treat analysis, and assessor blinding. Likewise, such studies should adhere to generally accepted standards for reporting clinical trials (e.g., the Consolidated Standards of Reporting Trials statement). To estimate the duration of TCE-induced improvements, long-term follow-up periods must be adopted in future studies. In addition, the long-term effectiveness of TCE for patients with chronic diseases must be estimated.

## Additional Information

**How to cite this article**: Wang, X. *et al.* Effect of traditional Chinese exercise on the quality of life and depression for chronic diseases: a meta-analysis of randomised trials. *Sci. Rep.* **5**, 15913; doi: 10.1038/srep15913 (2015).

## Supplementary Material

Supplementary Information

## Figures and Tables

**Table 1:** Characteristics of included studies.

Article,Year	Country/region	Participant Characteristic,Sample Size	Disease	Intervention/comparisongroups	Duration oftrial period	Outcomes	Timepoint
An (2008) 20	China	28 subjects (G1 = 14, G2 = 14). Mean age(SD): G1 = 65.4 y (8.2), G2 = 64.6 y (6.7)	Musculoskeletal disorder (Knee osteoarthritis)	G1: Baduanjin G2: No intervention	Five times a week for 8 weeks	Quality of life (SF-36)	8 weeks
Barrow (2007) 21	UK	65 subjects (G1 = 32, G2 = 33). Mean age: G1 = 68.4 y, G2 = 67.9 y	Cardiovascular diseases (Heart failure)	G1: Tai Chi G2: Usual care	Twice a week for 16 weeks	Depression (SCL-90)	16 weeks
Blake (2009) 22	UK	20 subjects (G1 = 10, G2 = 10). Mean age(SD): G1 = 46.2 y (11.27), G2 = 44.5 y (10.52)	Cardiovascular and cerebrovascular diseases (Brain injury)	G1: Tai Chi + Qigong G2: No intervention	Once a week for 8 weeks	Quality of life (GHQ)	8 weeks
Cai (2010) 23	China	60 subjects (G1 = 30, G2 = 30). Mean age(SD): G1 = 60.3 y (10.5), G2 = 61.3 y (7.4).	Cardiovascular and cerebrovascular diseases (Stroke)	G1: Baduanjin exercise + usual care G2: Usual care	Four or five times a week for 3 months	Quality of life (WHO QOL)	3 months
Chen (2008) 24	USA	162subjects(G1 = 57, G2 = 49) Mean age(SD): G1 = 61.3 y(8.4)G2 = 62.9 y(9.2)	Musculoskeletal disorder (Knee osteoarthritis)	G1: Qigong G2: Sham Qigong	Five or six sessions a week for 3 weeks	Depression (CES-D)	3 weeks 15 weeks
Chen (2013) 25	USA	96 subjects (G1 = 49, G2 = 47). Mean age(SD): G1 = 45.3 y(6.3), G2 = 44.7 y(9.7),	Cancer (Breast cancer)	G1: Qigong G2: Wait-list	Five times a week for 6 weeks	Depression (CES-D)	6 weeks 10 weeks 18 weeks
Cheung (2005) 26	Hong Kong	88 subjects (G1 = 47, G2 = 41). Mean age(SD): G1 = 57.2 y (9.5), G2 = 51.2 y (7.4)	Cardiovascular and cerebrovascular diseases (Hypertension)	G1: Qigong G2: Conventional exercise	Twice a week for 4 weeks	Quality of life (SF-36), depressing (CES-D)	4 weeks 8 weeks 16 weeks
Chyu (2010) 27	USA	61 subjects (G1 = 30, G2 = 31). Mean age(SD): G1 = 72.4 y (6.2), G2 = 71.3 y (6)	Musculoskeletal disorder (Osteopaenia)	G1: Tai Chi G2: No intervention	Three times a week for 24 weeks	Quality of life (SF-36)	12 weeks 24 weeks
Fransen (2007) 28	Australia	152 subjects (G1 = 56, G2 = 55, G3 = 41). Mean age(SD): G1 = 70.8 y (6.3), G2 = 70 y (6.3), G3 = 69.6 y (6.1)	Musculoskeletal disorder (Osteoarthritis)	G1: Tai Chi 2: HydrotherapyG3: No intervention	Twice a week for 12 weeks	Quality of life (SF-12)	6 weeks 12 weeks
Gemmell (2006) 29	New Zealand	18 subjects (G1 = 9, G2 = 9).	Cardiovascular and cerebrovascular diseases (Traumatic brain injury)	G1: Tai Chi G2: No intervention	Once a week for 6 weeks	Quality of life (SF-36)	6 weeks
Guan (2012) 30	China	80 subjects (G1 = 39, G2 = 40).Mean age(SD): G1 = 59.2 y (8.8), G2 = 58.7 y (8.3).	Diabetes	G1: Baduanjin + conventional treatment G2: Conventional treatment	Seven times a week for 4 months	Depression (SDS)	4 months
Haak (2008) 31	Sweden	57subjects(G1 = 29,G2 = 28) Mean age(SD): G1 = 54.0 y(9.4) G2 = 53.4 y(8.0)	Musculoskeletal disorder (Fibromyalgia Syndrome)	G1: Qigong G2: Waiting-list	7 weeks	Quality of life (WHOQOL), Depression (BDI)	4 months
Hart (2004) 32	Israel	152 subjects (G1 = 56, G2 = 55, G3 = 41). Mean age(SD): G1 = 70.8 y (6.3), G2 = 70 y (6.3), G3 = 69.6 y (6.1)	Cardiovascular and cerebrovascular diseases (Stroke)	G1: Tai Chi G2: Hydrotherapy G3: No intervention	Twice a week for 12 weeks	Quality of life (Duke Health Profile)	6 weeks 12 weeks
Ji (2012) 33	China	62 subjects (G1 = 32, G2 = 30). Mean age(SD): G1 = 60.31 y(7.23), G2 = 60.26 y(7.15)	Diabetes	G1: Baduanjin + drug G2: Drug	Once a day for 2 months	Depression (SDS)	2 months
Lam (2008) 34	Australia	53 subjects (G1 = 28, G2 = 25). Mean age(SD): G1 = 63.2 y (8.6), G2 = 60.7 y (12.2)	Diabetes (Type 2 diabetes)	G1: Tai Chi G3: No exercise	Twice a week for 6 months	Quality of life (SF-36)	6 months
Lee 2009) 35	Korea	44 subjects (G1 = 29, G2 = 15). Mean age(SD): G1 = 70.2 y (4.8), G2 = 66.9 y (6)	Musculoskeletal disorder (Osteoarthritis)	G1: Tai Chi and Qigong G2: No intervention	Twice a week for 8 weeks	Quality of life (SF-36)	8 weeks
Li (2010) 36	China	60 subjects (G1 = 30, G2 = 30). Age over 45 y	Musculoskeletal disorder (Osteoporosis)	G1: Tai Chi + usual care G2: Usual care	Once a day for 12 months	Quality of life (SF-36)	12 months
Li (2012) 37	China	68 subjects (G1 = 36, G2 = 32). Age range: 38 to 76 y	Cardiovascular and cerebrovascular diseases (Stroke)	G1: Tai Chi G2: Conventional exercise	Twice a week for 5 weeks	Depression (HAMD)	5 weeks
Li (2012) 38	China	70 subjects (G1 = 35, G2 = 35). Mean age(SD): G1 = 72.0 y (2.5), G2 = 73.0 y (3.0)	Chronic respiratory diseases (COPD)	G1: Tai Chi + respiratory exercise G2: Respiratory exercise	Once a day for 6 months	Depression (SCL-90)	6 months
Li (2013) 39	China	216 subjects (G1 = 54, G2 = 54, G3 = 54, G4 = 54). Mean age(SD): G1 = 50.42 y (9.68), G2 = 51.62 y (7.83), G3 = 54.21 y (9.47), G4 = 52.69 y (8.37)	Diabetes	G1: Baduanjin G2: Aerobic exercise G3: Tai Chi G4: Control	Once a day for 3 months	Quality of life (SF-36)	3 months 9 months
Liu (2012) 40	China	69 subjects (G1 = 33, G2 = 36). Mean age(SD): G1 = 62.64 y (5.98), G2 = 65.64 y (8.38),	Diabetes	G1: Baduanjin + education G2: Education	Once a week for 12 weeks	Depression (SDS)	6 weeks 12 weeks
Liu (2013) 41	Australia	41 subjects (G1 = 20, G2 = 21). Mean age(SD): G1 = 59 y (8), G2 = 59 y (8)	Diabetes	G1: Tai Chi G2: Usual medical-care	Three sessions per week for 12 weeks	Quality of life (SF-36)	12 weeks
Ng (2011) 42	Hong Kong	80 subjects (G1 = 40, G2 = 40). Mean age(SD): G1 = 71.75 y (1.05), G2 = 73.12 y (1.33)	Chronic respiratory diseases (COPD)	G1: Qigong G2: Conventional treatment	Four times a week for 6 month	Quality of life (SF-36)	3 months 6 months
Putiri (2012) 43	USA	32 subjects (G1 = 11, G2 = 11, G3 = 10). Mean age(SD): G1 = 57.0 y (6.3), G2 = 58.4 y (7.4), G3 = 59.4 y (6.8)	Diabetes (Type 2 diabetes)	G1: Qigong G2: Resistance training G3: Usual care	At least twice a week for 12 weeks	Depression (BDI)	12 weeks
Rendant (2011) 44	Germany	122 subjects (G1 = 42, G2 = 39, G3 = 41). Mean age(SD): G1 = 44.7 y (10.8), G2 = 44.4 y (10.9), G3 = 47.8 y (10.3)	Musculoskeletal disorder (Chronic neck pain)	G1: Qigong G2: Conventional exercise G3: Waiting List	1 session per week in the first 3 months, and biweeklysessions in the following 3 months	Quality of life (SF-36)	3 months 6 months
Robins (2013) 45	USA	145 subjects. aged 27–75 years	Cancer (Breast cancer)	G1: Tai Chi G2: Usual medical-care	90 minutes each week for a total of 10 weeks	Depression (CES-D)	3 months 4.5 months 6 months
Shen(2010) 46	USA	171 subjects (G1 = 42, G2 = 38, G3 = 44, G4 = 47). Mean age(SD): G1 = 58.3y(7.7), G2 = 57.6y(6.7), G3 = 57.6 y (7.5), G4 = 56.5 y (5.5)	Musculoskeletal disorder (Postmenopausal osteopenic women)	G1: Tai Chi + Placebo G2: Tai Chi + drug G3: Placebo G4: Drug	Three sessions a week for 12 weeks	Quality of life (SF-36)	8 weeks 12 weeks 16 weeks 24 weeks
Singh-Grewal (2007) 47	Canada	80 subjects (G1 = 41, G2 = 39). Mean age(SD): G1 = 11.7y (2.5), G2 = 11.5y (2.4)	Musculoskeletal disorder (Arthritis)	G1: Qigong G2: Aerobic training	Three times a week for 12-week	Quality of life (HRQOL)	12 weeks
Skoglund (2011) 48	Sweden	37 subjects. Age range: 42 to 54 y	Musculoskeletal disorder (Neck-shoulder pain)	G1: Qigong G2: No Intervention	Four times a week for six weeks	Quality of life (SF-12)	6 weeks
Sprod (2012) 49	USA	65 subjects (G1 = 32, G2 = 33). Mean age: G1 = 68.4 y, G2 = 67.9 y	Cancer (Breast cancer)	G1: Tai Chi G2: Usual care	Three times a week for 12 weeks	Quality of life (SF-36)	12 weeks
Stephens (2008) 50	Canada	30 subjects (G1 = 16, G2 = 14). Mean age(SD): G1 = 12.9 y(2.7), G2 = 13.6 y(1.8)	Musculoskeletal disorder (Fibromyalgia)	G1: Qigong G2: Aerobic training	Three times a week for 12 weeks	Depression (Chinldhood depression inventory)	12 weeks
Taylor-Piliae (2012) 51	USA	20 subjects (G1 = 10, G2 = 10). Mean age(SD): G1 = 46.2 y (11.27), G2 = 44.5 y (10.52)	Cardiovascular and cerebrovascular diseases (Chronic stroke)	G1: Tai Chi G2: Usual care	Once a week for 8 weeks	Quality of life (SF-36), depressing (CES-D)	8 weeks
Trott (2009) 52	Germany	117 subjects (G1 = 38, G2 = 39, G3 = 40). Mean age(SD): G1 = 75.9 y(7.6), G2 = 76.0 y(7.2) G3 = 75.7 y(7.6)	Musculoskeletal disorder (Chronic neck pain)	G1: Qigong G2: General exercise G3: Wait-list	2 sessions a week for 3 months	Quality of life(SF-36)	3 months 6 months
Tsang (2003) 53	Hong Kong	50 subjects (G1 = 24, G2 = 26). Mean age(SD): G1 = 72.93 y(9.53), G2 = 76.27 y(8.40)	Chronic physical illnesses	G1: Qigong G2: Usual care	Twice a week for 12-week	Quality of life (WHOQOL)	6 weeks 12 weeks
Tsang (2007) 54	Australia	38 subjects (G1 = 18, G2 = 20). Mean age(SD): G1 = 66 y (8), G2 = 65 y (8)	Diabetes	G1: Tai Chi G2: Sham Tai Chi	Twice a week for 16 weeks	Quality of life (SF-36)	16 weeks
Wang (2008) 55	USA	20 subjects (G1 = 10, G2 = 10). Mean age(SD): G1 = 48 y (10), G2 = 51 y (17)	Musculoskeletal disorder (Rheumatoid arthritis)	G1: Tai Chi G2: Stretching + education	Twice a week for 12 weeks	Quality of life (SF-36), depressing (CES-D)	12 weeks
Wang (2010) 56	USA	66 subjects (G1 = 33, G2 = 33). Mean age(SD): G1 = 49.7 y (11.8), G2 = 50.5 y (10.5)	Musculoskeletal disorder (fibromyalgia)	G1: Tai Chi G2: Stretching + education	Twice a week for 12 weeks	Quality of life (SF-36), depressing (CES-D)	12 weeks 24 weeks
Wang (2010) 57	Japan	34 subjects (G1 = 17, G2 = 17). Mean age(SD): G1 = y (), G2 = y ()	Cardiovascular and cerebrovascular diseases (Cerebral vascular disorder)	G1: Tai Chi G2: General exercise	Once a week for 12 weeks	Quality of life (GHQ)	12 weeks
Wang (2009) 58	USA	40 subjects (G1 = 20, G2 = 20). Mean age(SD): G1 = 63 y (8.1), G2 = 68 y (7.0)	Musculoskeletal disorder (Knee osteoarthritis)	G1: Tai Chi G2: Stretching + education	Twice a week for 12 weeks	Quality of life(SF-36),depression (CES-D)	12 weeks 24 weeks 48 weeks
Wang (2009) 59	China	64 subjects (G1 = 34, G2 = 30). Mean age(SD): G1 = 48.24 y (10.06), G2 = 47.86 y (11.12)	Diabetes (Type 2 diabetes)	G1: Tai Chi + drug G2: Drug	Five or seven times a week for 6 months	Quality of life (SF-36)	6 months
Wang (2010) 60	China	120 subjects (G1 = 58, G2 = 62). Age range: 28–65 y	Cancer (Breast cancer)	G1: Tai Chi G2: Conventional exercise	Twice a day for 170 days	Quality of life (WHOQ OL)	170 days
Wang (2012)^61^	China	69 subjects (G1 = 36, G2 = 33). Mean age(SD): G1 = 55.8 y (3.54), G2 = 51.2 y (7.8)	Cardiovascular and cerebrovascular diseases (Stroke)	G1: Tai Chi G2: Conventional exercise	Twice a week for 3 months	Depression (HAMD)	6 months
Wang (2013) 62	China	60 subjects (G1 = 30, G2 = 30). Mean age(SD): G1 = 55.25 y (11.13), G2 = 54.86 y (12.05)	Cardiovascular and cerebrovascular diseases	G1: Tai Chi + usual care G2: Usual care	Five times a week for 6 months	Quality of life (SF-36)	3 months 6 months
Wayne (2012) 63	USA	86 subjects (G1 = 43, G2 = 43). Mean age(SD): G1 = 58.8 y (5.6), G2 = 60.4 y (5.3)	Musculoskeletal disorder (Post-menopausal osteopenic)	G1: Tai Chi exercise G2: Usual care	99.5 hours over the 9 month	Quality of life (SF-36)	12 weeks
Wei (2014) 64	China	60 subjects (G1 = 20, G2 = 20, G3 = 20). Mean age(SD): G1 = 63.9y (7.6), G2 = 64.8y (5.8), G3 = 65.3 y (6.0)	Diabetes (Type 2 diabetes)	G1: Baduanjin G2: Walking G3: Control	Five times a week for 3 months	Quality of life (SF-36)	3 months
Wenneberg (2004) 65	Sweden	31 subjects (G1 = 16, G2 = 15).	Musculoskeletal disorder (Muscular dystrophy)	G1: Qigong G2: No intervention	Once a week for two months	Quality of life (SF-36)	2 months
Wu (1999) 66	USA	22 subjects (G1 = 11, G2 = 11). Mean age(SD): G1 = 37.8 y(11.7), G2 = 39.3 y(13.2)	Musculoskeletal disorder (Pain syndrome type 1)	G1: Qigong G2: Sham Qigong	Twice a week for 3 weeks, everyday for the following 7 weeks	Depression (BDI)	1 weeks 3 weeks 6 weeks 10 weeks
Wu (2012) 67	China	52 subjects (G1 = 26, G2 = 26). Mean age(SD): G1 = 55.92 y (9.25), G2 = 56.46 y (9.13).	Musculoskeletal disorder (Low back pain)	G1: Baduanjin + electrotherapy G2: Electrotherapy	Four or five times a week for 1 month	Quality of life (SF-36)	1 month
Yang (2005) 68	Korea	43 subjects (G1 = 20, G2 = 23). Mean age(SD): G1 = 72.58 y (5.41), G2 = 72.67 y (7.49)	Musculoskeletal disorder (Chronic pain)	G1: Qi gong exercise G2: Usual care	Twice a week for four weeks	Depression (POMS)	1 weeks 2 weeks 3 weeks 4 weeks 6 weeks
Yeh (2010) 69	USA	10 subjects (G1 = 5, G2 = 5). Mean age(SD): G1 = 65 y (6), G2 = 66 y (6)	Chronic respiratory diseases (COPD)	G1: Tai Chi + usual care G2: Usual care	Twice a week for 12 weeks	Depression (CES-D)	12 weeks
Yeh (2011) 70	USA	100 subjects (G1 = 50, G2 = 50). Mean age(SD): G1 = 68.1 y (11.9), G2 = 66.6 y (12.1)	Cardiovascular and cerebrovascular diseases (Chronic heart failure)	G1: Tai Chi G2: Education	Twice a week for 12 weeks	Depression (POMS-D)	12 weeks
Yeh (2013) 71	USA	16 subjects (G1 = 8, G2 = 8). Mean age(SD): G1 = 68 y (11), G2 = 63 y (11)	Cardiovascular and cerebrovascular diseases (Heart failure)	G1: Tai Chi G2: Aerobic exercise	Twice a week for 12 weeks	Depression (POMS-D)	12 weeks
Zhou (2014) 72	China	25 subjects (G1 = 13, G2 = 12). Age range: 58-80 y	Diabetes	G1: Qigong G2: Education	Once a week for 12 weeks	Depression (SDS)	12 weeks
Wang (2014) 73	China	60 subjects (G1 = 30, G2 = 30). Mean age(SD): G1 = 72.9 y (9.09), G2 = 71.1 y (8.4)	Chronic respiratory diseases (COPD)	G1: Liuzijue G2: Conventional therapy	Seven times a week for 12 weeks	Depression (HAMD)	12 weeks
Wang (2014) 74	China	70 subjects (G1 = 35, G2 = 35). Mean age(SD): G1 = 67.8 y (6.6), G2 = 68.0 y (7.5)	Diabetes (Type 2 diabetes)	G1: Taichi G2: Conventional therapy	Five times a week for 8 weeks	Depression (SCL-90)	8 weeks
Sun (2014) 75	China	80 subjects (G1 = 38, G2 = 42). Mean age(SD): G1 = 68.1 y (4.4), G2 = 69.1 y (4.2)	Cardiovascular and cerebrovascular diseases	G1: Taichi G2: Education	Seven times a week for 8 weeks	Depression (SDS)	8 weeks
Meng (2014) 76	China	200 subjects (G1 = 100, G2 = 100). Age range: 60–89 year	Diabetes (Type 2 diabetes)	G1: Taichi G2: Conventional exercise	Four times a week for 12 weeks	Quality of life (SF-36)	8 weeks
Fang (2014) 77	China	89 subjects (G1 = 30, G2 = 29, G3 = 30). Mean age(SD): G1 = 56.6 y (8.85), G2 = 58.2 y (8.9), G3 = 57.1 y (9.2)	Diabetes	G1: Qigong + education G2: Walk + educationG3: Education	Five times a week for 12 weeks	Depression (SCL-90)	12 weeks
Taylor (2014) 78	USA	145 subjects (G1 = 53, G2 = 44, G3 = 48). Mean age(SD): G1 = 71.5 y (10.3), G2 = 69.6 y (9.4), G3 = 68.2 y (10.3)	Cardiovascular and cerebrovascular diseases (Stroke)	G1: Tai Chi G2: Strength exercise G3: Usual care	Three times a week for 12 weeks	Quality of life (SF-36) and depression (CES-D)	12 weeks
Park (2014) 79	Korea	40 subjects (G1 = 19, G2 = 21). Mean age(range): G1 = 52 y (43–61), G2 = 54 y (45–62)	Cardiovascular and cerebrovascular diseases	G1: Qigong G2: No intervention	Three times a week for 8 weeks	Quality of life (SF-36)	4 weeks 8 weeks

BDI: Beck Depression Inventory, CES-D: Center for Epidemiologic Studies Depression Scale, COPD: Chronic obstructive pulmonary disease, GHQ: General Health Questionnaire, HAMD: Hamilton Depression Scale, POMS-D: Profile of Mood States-Depression, SCL-90: Symptom Checklist 90, SDS: Self-rating depression scale, SF-36: Short-form 36, WHOQOL: World Health Organization Quality of Life.

**Table 2:** Risk of bias assessment of included studies.

Article(Year)	Randomallocation	Concealedallocation	Baselinecomparability	Blindsubjects	Blindtherapists	Blindassessors	Adequatefollow-up	Intention totreat analysis	Between-groupcomparisons	Point estimatesand variability
An (2008) 20	Yes	No	Yes	No	No	No	No	No	Yes	Yes
Barrow (2007) 21	Yes	No	Yes	No	No	No	No	No	Yes	Yes
Blake (2009) 22	Yes	No	Yes	No	No	No	Yes	Yes	Yes	Yes
Cai (2010) 23	Yes	No	Yes	No	No	No	No	No	Yes	Yes
Chen (2008) 24	Yes	No	Yes	No	No	Yes	No	Yes	Yes	Yes
Chen (2013) 25	Yes	No	Yes	No	No	No	Yes	No	Yes	Yes
Cheung (2005) 26	Yes	No	Yes	No	No	No	No	Yes	Yes	Yes
Chyu (2010) 27	Yes	No	Yes	No	No	Yes	Yes	No	Yes	Yes
Fransen (2007) 28	Yes	Yes	Yes	No	No	Yes	Yes	Yes	Yes	Yes
Gemmell (2006) 29	Yes	No	Yes	No	No	Yes	No	No	Yes	Yes
Guan (2012) 30	Yes	No	Yes	No	No	No	No	No	Yes	Yes
Haak (2008) 31	Yes	No	Yes	No	No	No	Yes	No	Yes	Yes
Hart (2004) 32	Yes	No	No	No	No	Yes	No	No	Yes	Yes
Ji (2012) 33	Yes	No	Yes	No	No	Yes	No	No	Yes	Yes
Lam (2008) 34	Yes	No	Yes	No	No	Yes	Yes	No	Yes	Yes
Lee (2009) 35	Yes	Yes	Yes	No	No	Yes	Yes	Yes	Yes	Yes
Li (2010) 36	Yes	No	No	No	No	No	No	No	Yes	Yes
Li (2012) 37	Yes	No	Yes	No	No	Yes	No	No	Yes	Yes
Li (2012) 38	Yes	No	Yes	No	No	No	No	No	Yes	Yes
Li (2013) 39	Yes	No	Yes	No	No	No	No	No	Yes	Yes
Liu (2012) 40	Yes	No	Yes	No	No	No	No	No	Yes	Yes
Liu (2013) 41	Yes	No	No	No	No	No	No	Yes	Yes	Yes
Ng (2011) 42	Yes	Yes	Yes	No	No	Yes	No	Yes	Yes	Yes
Putiri (2012) 43	Yes	No	Yes	No	No	No	No	No	Yes	Yes
Rendant (2011) 44	Yes	Yes	Yes	No	No	No	Yes	Yes	Yes	Yes
Robins (2013) 45	yes	No	Yes	No	No	No	Yes	No	Yes	Yes
Shen (2010) 46	Yes	No	Yes	No	No	Yes	Yes	Yes	Yes	Yes
Singh-Grewal (2007) 47	Yes	Yes	Yes	No	No	Yes	Yes	Yes	Yes	Yes
Skoglund (2011) 48	Yes	No	Yes	No	No	No	Yes	No	Yes	Yes
Sprod (2012) 49	Yes	Yes	Yes	No	No	No	No	No	Yes	Yes
Stephens (2008) 50	Yes	Yes	Yes	No	No	Yes	No	Yes	Yes	Yes
Taylor-Piliae (2012) 51	Yes	Yes	Yes	No	No	Yes	Yes	No	Yes	Yes
Trott (2009) 52	Yes	No	Yes	No	No	No	No	Yes	Yes	Yes
Tsang (2003) 53	Yes	No	Yes	No	No	No	No	No	Yes	Yes
Tsang (2007) 54	Yes	Yes	Yes	No	No	No	Yes	Yes	Yes	Yes
Wang (2008) 55	Yes	Yes	Yes	No	No	Yes	Yes	Yes	Yes	Yes
Wang (2010) 56	Yes	Yes	Yes	No	No	No	Yes	Yes	Yes	Yes
Wang (2010) 57	Yes	No	Yes	No	No	Yes	Yes	Yes	Yes	Yes
Wang (2009) 58	Yes	Yes	Yes	No	No	No	Yes	Yes	Yes	Yes
Wang (2009) 59	Yes	No	Yes	No	No	No	No	No	Yes	Yes
Wang (2010) 60	Yes	No	Yes	No	No	No	Yes	No	Yes	Yes
Wang (2012)^61^	Yes	No	Yes	No	No	No	No	No	Yes	Yes
Wang (2013) 62	Yes	No	Yes	No	No	No	Yes	No	Yes	Yes
Wayne (2012) 63	Yes	No	Yes	No	No	Yes	Yes	Yes	Yes	Yes
Wei (2014) 64	Yes	No	Yes	No	No	No	No	No	Yes	Yes
Wenneberg (2004) 65	Yes	No	Yes	No	No	Yes	Yes	No	Yes	Yes
Wu (1999) 66	Yes	No	Yes	No	No	No	Yes	No	Yes	Yes
Wu (2012) 67	Yes	No	Yes	No	No	No	No	No	Yes	Yes
Yang (2005) 68	Yes	No	Yes	No	No	No	Yes	No	Yes	Yes
Yeh (2010) 69	Yes	No	Yes	No	No	Yes	Yes	Yes	Yes	Yes
Yeh (2011) 70	Yes	No	Yes	No	No	Yes	Yes	Yes	Yes	Yes
Yeh (2013) 71	Yes	No	Yes	No	No	Yes	No	Yes	Yes	Yes
Zhou (2014) 72	Yes	No	Yes	No	No	No	No	No	Yes	Yes
Wang (2014) 73	Yes	No	Yes	No	No	No	No	No	Yes	Yes
Wang (2014) 74	Yes	No	Yes	No	No	No	No	No	Yes	Yes
Sun (2014) 75	Yes	No	Yes	No	No	No	No	No	Yes	Yes
Meng (2014) 76	Yes	No	Yes	No	No	No	No	No	Yes	Yes
Fang (2014) 77	Yes	Yes	Yes	No	No	No	No	No	Yes	Yes
Taylor (2014) 78	Yes	Yes	Yes	No	No	Yes	No	Yes	Yes	Yes
Park (2014) 79	Yes	Yes	Yes	No	No	Yes	Yes	No	Yes	Yes

**Table 3:** Summary of results.

Outcome	Trials	Participants	Statistical Method	Effect Estimate	Heterogeneity	P value
Short term						
Quality of life						
SF-36 total	6 35,39,49,62,67,76	591	Std. Mean Difference (IV, Random, 95% CI)	0.59 [0.32, 0.87]	0.05	<0.001
SF-36 physical function	19 27,29,35,41,44,46,49,51,52,54,55,56,58,63,64,65,76,78,79	1332	Std. Mean Difference (IV, Random, 95% CI)	0.35 [0.13, 0.56]	<0.001	0.002
SF-36 mental health	19 20,27,29,35,44,46,49,51,52,54,55,56,58,63,64,65,76,78,79	1312	Std. Mean Difference (IV, Random, 95% CI)	0.28 [0.11, 0.46]	<0.001	0.002
SF-36 general health	11 20,27,29,41,46,49,54,55,65,76,79	648	Std. Mean Difference (IV, Random, 95% CI)	0.15 [−0.00, 0.31]	0.66	0.06
GHQ	2 22,57	49	Std. Mean Difference (IV, Random, 95% CI)	−0.68 [−1.26, −0.09]	0.73	0.02
WHO-QOL physical health	4 23,31,53,60	287	Std. Mean Difference (IV, Random, 95% CI)	0.13 [−0.59, 0.85]	<0.001	0.73
WHO-QOL psychological health	4 23,31,53,60	287	Std. Mean Difference (IV, Random, 95% CI)	0.22 [−0.04, 0.47]	0.32	0.09
WHO-QOL general health	4 23,31,53,60	287	Std. Mean Difference (IV, Random, 95% CI)	0.68 [0.04, 1.32]	<0.001	0.04
Depression						
CES-D	8 24,25,51,55,56,58,69,78	508	Std. Mean Difference (IV, Random, 95% CI)	−0.86 [−1.42, −0.31]	<0.001	0.002
SDS	5 30,33,40,72,75	315	Std. Mean Difference (IV, Random, 95% CI)	−0.6 [−0.83, −0.36]	0.37	<0.001
BDI	3 26,31,43	180	Std. Mean Difference (IV, Random, 96% CI)	−0.15 [−0.75, 0.44]	0.03	0.61
POMS	3 68,70,71	156	Std. Mean Difference (IV, Random, 95% CI)	−1.64 [−2.55, −0.73]	<0.001	<0.001
HAMD	3 37,61,73	189	Std. Mean Difference (IV, Random, 96% CI)	−1.36 [−1.97, −0.75]	0.03	<0.001
Mid term						
Quality of life						
SF-36 total	2 39,62	276	Std. Mean Difference (IV, Random, 95% CI)	0.61 [0.16, 1.05]	0.12	0.008
SF-36 physical function	9 26,27,34,44,46,52,56,58,59	771	Std. Mean Difference (IV, Random, 95% CI)	0.49 [0.12, 0.85]	<0.001	0.009
SF-36 mental health	10 27,34,42,44,46,52,56,58,59,63	846	Std. Mean Difference (IV, Random, 95% CI)	0.39 [0.08, 0.71]	<0.001	0.02
SF-36 general health	7 26,27,34,42,46,59,63	592	Std. Mean Difference (IV, Random, 95% CI)	0.05 [−0.24, 0.34]	0.007	0.73
Depression						
CES-D	4 24,25,56,58	308	Std. Mean Difference (IV, Random, 95% CI)	−0.41 [−0.64, −0.18]	0.78	<0.001
SCL-90	4 21,28,74,77	284	Std. Mean Difference (IV, Random, 95% CI)	−0.7 [−1.32, −0.08]	<0.001	0.03

BDI: Beck Depression Inventory, CES-D: Center for Epidemiologic Studies Depression Scale, GHQ: General Health Questionnaire, HAMD: Hamilton Depression Scale, POMS-D: Profile of Mood States-Depression, SCL-90: Symptom Checklist 90, SDS: Self-rating depression scale, SF-36: Short-form 36, WHOQOL: World Health Organization Quality of Life.
