# Supplementary material for: Effect of traditional Chinese exercise on the quality of life and depression for chronic diseases: a meta-analysis of randomised trials
Source: Sci Rep. 2015 Nov 3;5:15913. doi: 10.1038/srep15913 (PMC4630632; doi:10.1038/srep15913)
Supplement: Supplementary Information [file srep15913-s1.pdf]

Supplementary Material

Effect of traditional Chinese exercise on the quality of life and depression for chronic diseases: a meta-analysis of randomised trials

Xueqiang Wang [a b]+, Yanling Pi [c]+, Binglin Chen [a], Peijie Chen [a] ✉, Yu Liu [d], Ru Wang [d], Xin Li [a], Yi Zhu [e], Yujie Yang [e], Zhanbin Niu [d]

a Department of Sport Rehabilitation, Shanghai University of Sport, Shanghai, China; b Department of Rehabilitation Medicine, Shanghai Shangti Orthopaedic Hospital, Shanghai, China; c Department of Rehabilitation Medicine, Shanghai Punan Hospital, Shanghai, China; d Key Laboratory of Exercise and Health Sciences of Ministry of Education, Shanghai University of Sport, Shanghai, China; e Second School of Clinical Medical, Nanjing University of Chinese Medicine, Nanjing, China.

Correspondence to: Prof. Peijie Chen, chenpeijie@sus.edu.cn. Department of Sport Rehabilitation, Shanghai University of Sport, 399 Changhai RD, Shanghai, 200438, China.

Supplementary Figure S1

Study or Subgroup	TCE Mean	TCE SD	TCE Total	Control Mean	Control SD	Control Total	Weight	Mean Difference IV, Random, 95% CI
**Musculoskeletal disorder**								
An (2008) 20	7.8	14.69	11	-0.5	11.74	10	1.4%	8.30 [-3.03, 19.63]
Chyu (2010) 27	2.27	9.32	27	-0.03	7.54	29	6.8%	2.30 [-2.16, 6.76]
Lee (2009) 35	19.2	15.9	29	9.1	10.3	15	2.8%	10.10 [2.31, 17.89]
Rendant (2011) 44	6.9	9.41	42	-1.31	10.57	80	8.9%	8.21 [4.54, 11.88]
Shen(2010) 46	2.9	16.2	80	-1.15	14.54	91	6.5%	4.05 [-0.59, 8.69]
Trott (2009) 52	2	10.37	38	-0.25	11.97	79	7.4%	2.25 [-1.97, 6.47]
Wang (2008) 55	7	18.6	10	2	6.6	10	1.2%	5.00 [-7.23, 17.23]
Wang (2009) 58	2.14	10.26	20	1.93	10.26	20	3.9%	0.21 [-6.15, 6.57]
Wang (2010) 56	7.7	11.28	33	1.6	11.14	33	5.1%	6.10 [0.69, 11.51]
Wayne(2012) 63	0.1	9.95	43	-1.2	5.74	43	9.7%	1.30 [-2.13, 4.73]
Wenneberg (2004) 65	0	19.09	16	4	19.91	15	1.0%	-4.00 [-17.75, 9.75]
**Cardiovascular diseases**								
Gemmell (2006) 29	-1.72	21.71	9	10.25	19.94	9	0.5%	-11.97 [-31.23, 7.29]
Park (2014) 79	8.68	22.23	19	0	18.64	21	1.1%	8.68 [-4.11, 21.47]
Taylor (2014) 78	3.1	11.62	53	3	10.23	92	8.6%	0.10 [-3.66, 3.86]
Taylor-Piliae (2012) 51	5.6	10.57	13	0.8	12.39	12	2.1%	4.80 [-4.26, 13.86]
**Cancer**								
Sprod (2012) 49	2.44	0.84	9	0.1	0.53	10	23.4%	2.34 [1.70, 2.98]
**Diabetes**								
Meng (2014) 76	4.79	15.57	100	1.66	16.17	100	7.0%	3.13 [-1.27, 7.53]
Tsang (2007) 54	-0.27	15.304	15	4.5	11.855	16	1.9%	-4.77 [-14.45, 4.91]
Wei (2014) 64	8.14	4.5	20	1.3	51.07	40	0.7%	6.84 [-9.11, 22.79]
**Total (95% CI)**			**587**			**725**	**100.0%**	**3.05 [1.68, 4.43]**

Heterogeneity: Tau² = 1.99; Chi² = 25.75, df = 18 (P = 0.11); I² = 30%
Test for overall effect: Z = 4.36 (P < 0.0001)
Test for subgroup differences: Chi² = 2.57, df = 3 (P = 0.46), I² = 0%

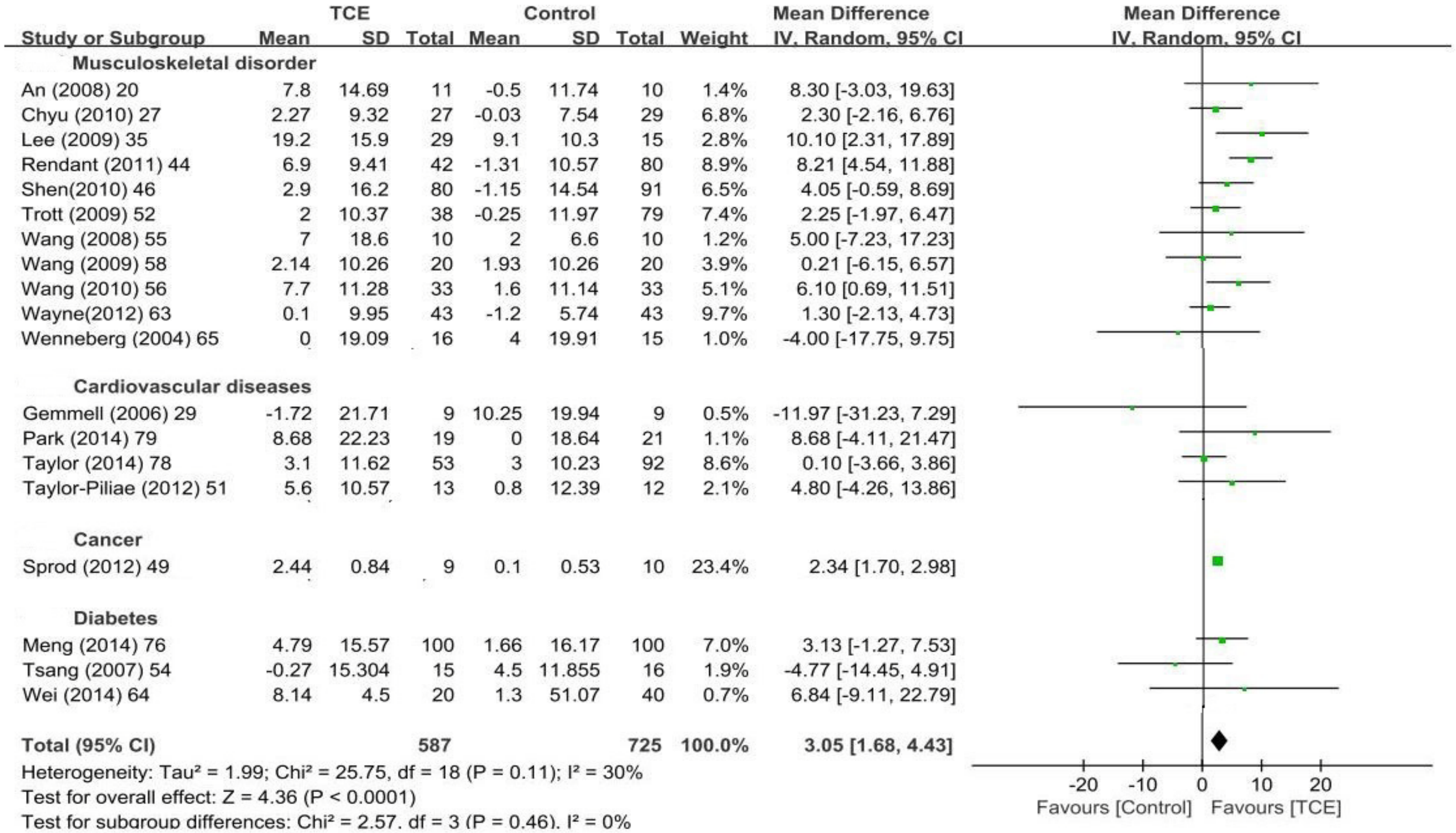

Supplementary Figure S1: Meta-analyses of traditional Chinese exercises on short form-36 mental health at the short term. SD=standard deviation; 95% CI=95% confidence intervals; IV=inverse variance.

Supplementary Figure S2

Study or Subgroup	TCE Mean	TCE SD	TCE Total	Control Mean	Control SD	Control Total	Weight	Mean Difference IV, Random, 95% CI
**Musculoskeletal disorder**								
Chyu (2010) 27	1.78	9.73	26	0.03	7.77	28	10.6%	1.75 [-2.97, 6.47]
Rendant (2011) 44	1.4	11.08	42	-0.49	10.48	80	12.1%	1.89 [-2.17, 5.95]
Shen(2010) 46	4.76	14.86	80	-2.94	15.38	91	11.0%	7.70 [3.16, 12.24]
Trott (2009) 52	-3.3	10.99	38	-4.81	11.33	79	11.5%	1.51 [-2.79, 5.81]
Wang (2010) 56	8.5	4.34	33	1.2	0.59	33	18.5%	7.30 [5.81, 8.79]
Wang（2009）59	4.07	17.76	34	1.41	16.19	30	5.3%	2.66 [-5.66, 10.98]
Wayne(2012) 63	0.6	8.9	43	-1.8	7.83	43	13.3%	2.40 [-1.14, 5.94]
**Chronic respiratory diseases**								
Ng (2011) 42	0.1	18.38	40	-3.1	17.22	40	5.8%	3.20 [-4.61, 11.01]
**Diabetes**								
Lam (2008) 34	2.25	19.71	24	-1.43	11.89	22	4.4%	3.68 [-5.64, 13.00]
Wang (2009) 58	4.39	10.27	20	4.5	10.26	20	7.6%	-0.11 [-6.47, 6.25]
**Total (95% CI)**			**380**			**466**	**100.0%**	**3.58 [1.36, 5.80]**

Heterogeneity: $Tau^2 = 6.37$; $Chi^2 = 21.96$, df = 9 (P = 0.009); $I^2 = 59\%$
Test for overall effect: Z = 3.16 (P = 0.002)
Test for subgroup differences: $Chi^2 = 0.90$, df = 2 (P = 0.64), $I^2 = 0\%$

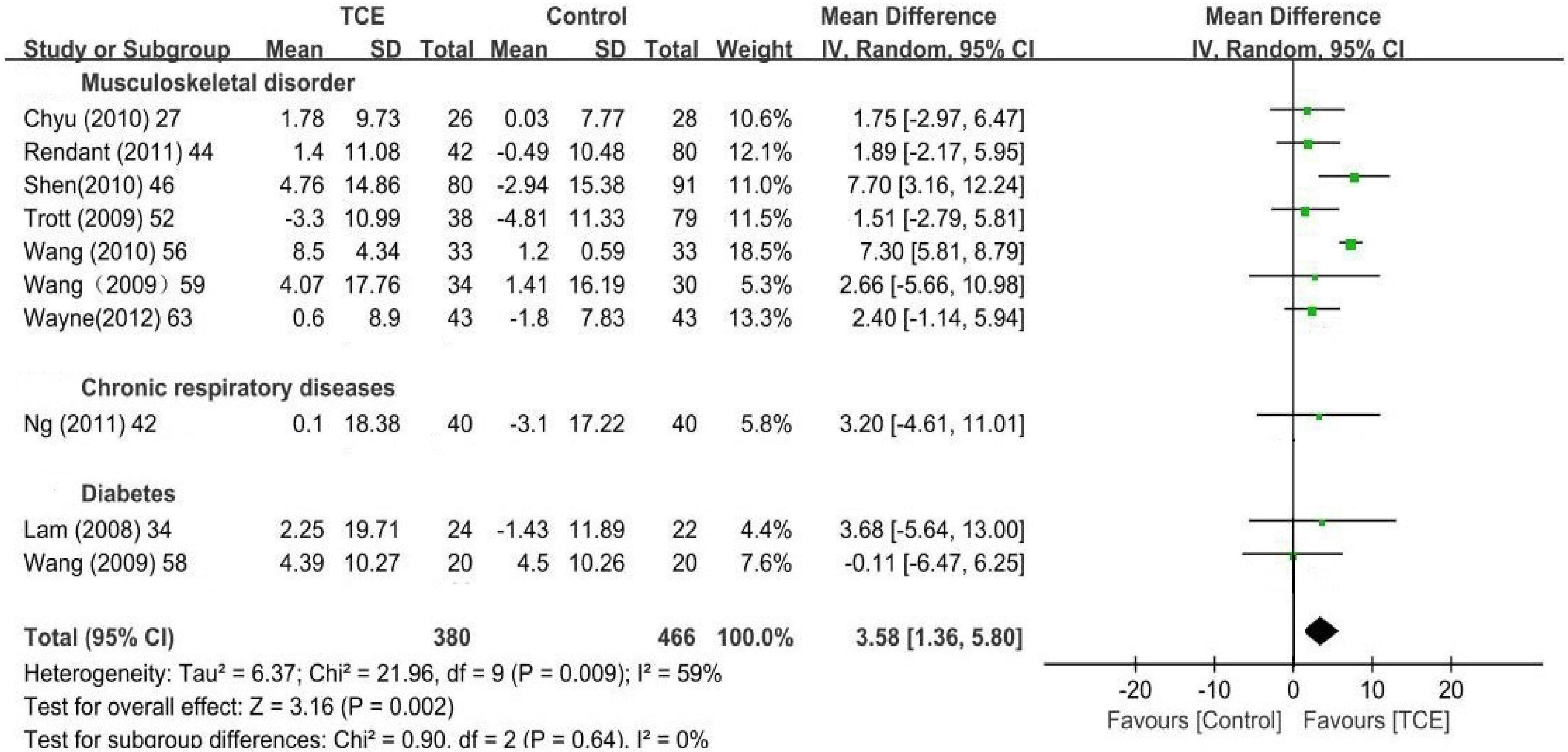

Supplementary Figure S2: Meta-analyses of traditional Chinese exercises on short form-36 mental health at the min-term. SD=standard deviation; 95% CI=95% confidence intervals; IV=inverse variance.

Supplementary Figure S3:

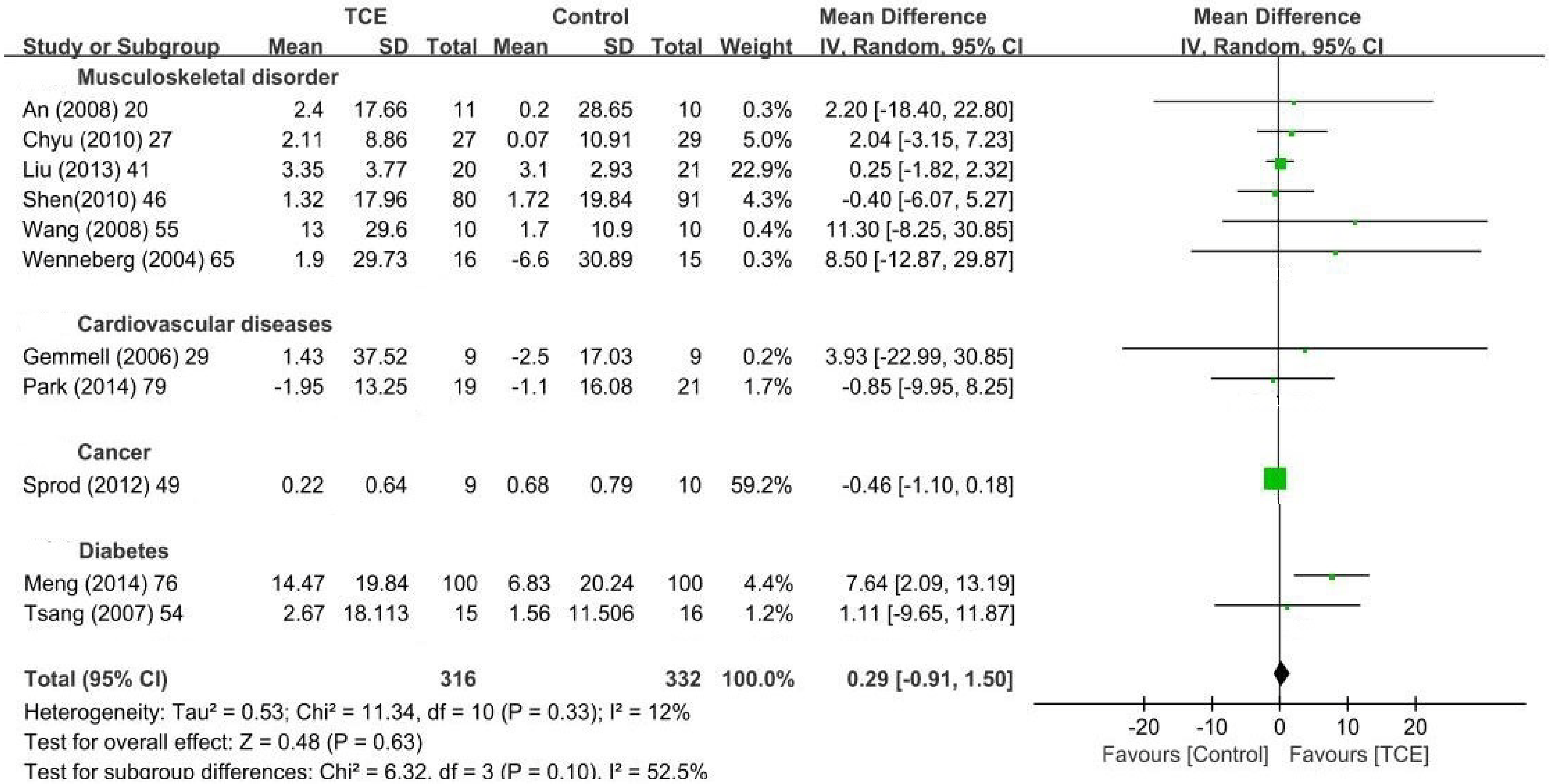

Supplementary Figure S3: Meta-analyses of traditional Chinese exercises on short form-36 general health at the short term. SD=standard deviation; 95% CI=95% confidence intervals; IV=inverse variance.

Supplementary Figure S4

Study or Subgroup	TCE Mean	TCE SD	TCE Total	Control Mean	Control SD	Control Total	Weight	Mean Difference IV, Random, 95% CI
**Musculoskeletal disorder**								
Chyu (2010) 27	3.38	8.7	26	-0.79	11.07	28	13.6%	4.17 [-1.12, 9.46]
Shen(2010) 46	1.15	17.95	80	0.91	19.92	91	12.3%	0.24 [-5.44, 5.92]
Wayne(2012) 63	-0.9	7.67	43	-0.1	6.6	43	24.0%	-0.80 [-3.82, 2.22]
**Cardiovascular diseases**								
Cheung (2005) 26	3.8	2	47	5.4	2.5	44	36.2%	-1.60 [-2.53, -0.67]
**Diabetes**								
Lam (2008) 34	6.97	27.35	24	2.27	20.09	22	2.9%	4.70 [-9.09, 18.49]
Wang（2009）59	13.06	23.34	34	3.09	25.11	30	3.7%	9.97 [-1.96, 21.90]
**Chronic respiratory diseases**								
Ng (2011) 42	1.4	17.66	40	-4	19.17	40	7.3%	5.40 [-2.68, 13.48]
**Total (95% CI)**			**294**			**298**	**100.0%**	**0.72 [-1.70, 3.15]**

Heterogeneity: $Tau^2 = 3.99$; $Chi^2 = 11.61$, df = 6 (P = 0.07); $I^2 = 48\%$
Test for overall effect: Z = 0.58 (P = 0.56)
Test for subgroup differences: $Chi^2 = 8.58$, df = 3 (P = 0.04), $I^2 = 65.0\%$

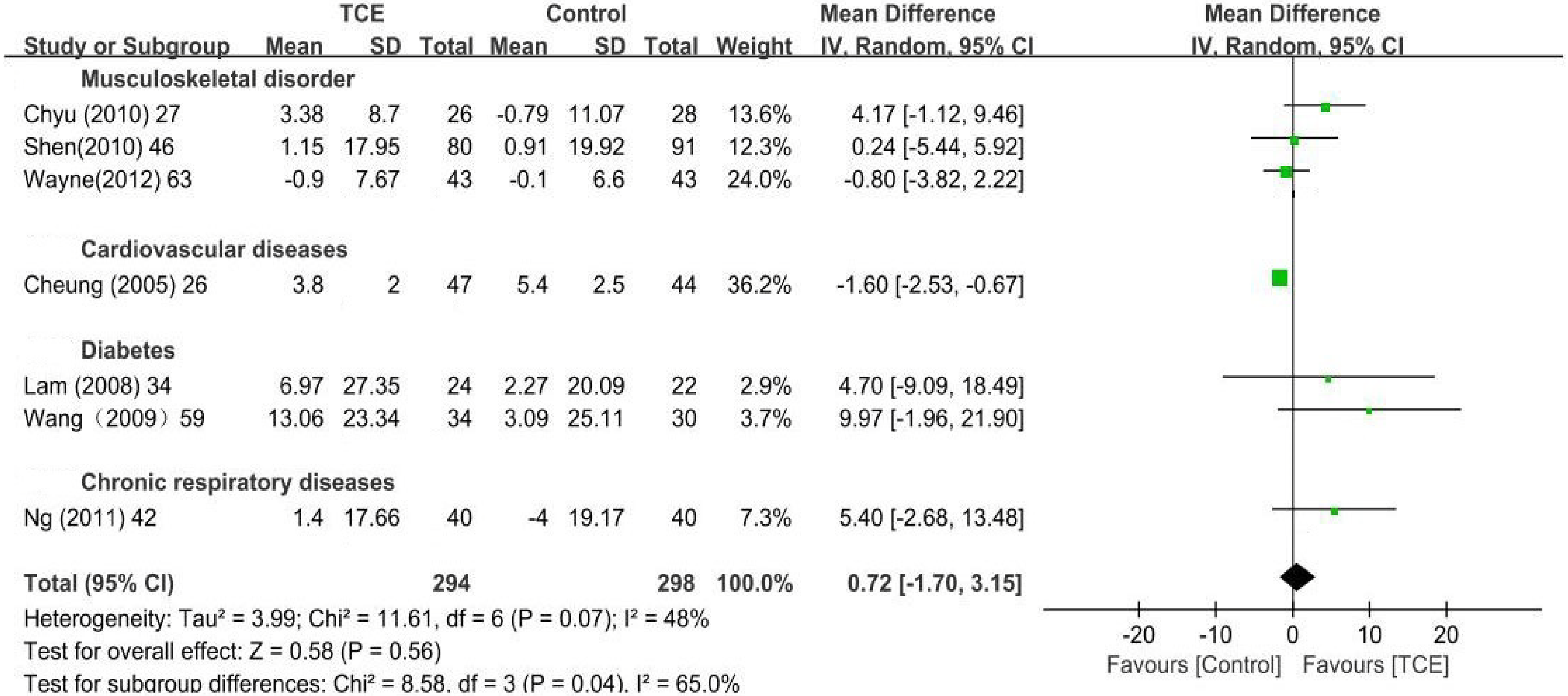

Supplementary Figure S4: Meta-analyses of traditional Chinese exercises on short form-36 general health at the mid-term. SD=standard deviation; 95% CI=95% confidence intervals; IV=inverse variance.

Supplementary Figure S5

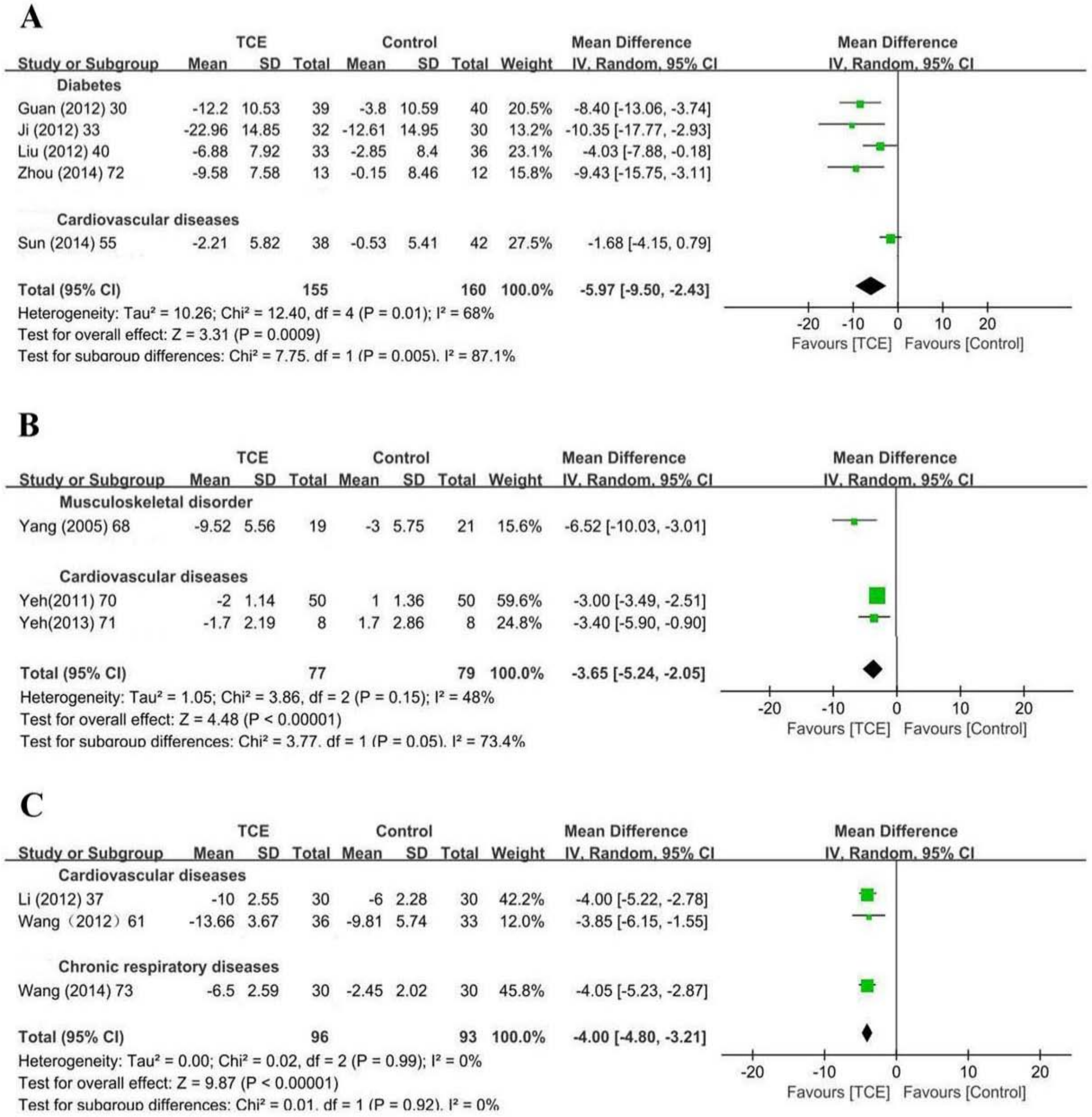

Supplementary Figure S5: Meta-analyses of traditional Chinese exercises on Self-rating depression scale (A), Profile of Mood States-depression (B), Hamilton Depression Scale (C) at the short term. SD=standard deviation; 95% CI=95% confidence intervals; IV=inverse variance.

Supplementary Table S1: Search strategies for all databases.

1. Search Strategy for PubMed:

#1 "Single-Blind Method"[Mesh] OR "Double-Blind Method"[Mesh] OR "Randomized Controlled Trials as Topic"[Mesh] OR "Randomized Controlled Trial" [Publication Type] OR "Intention to Treat Analysis"[Mesh] OR "Controlled Clinical Trials as Topic"[Mesh] OR "Clinical Trials as Topic"[Mesh] OR "Clinical Trial" [Publication Type] OR randomized controlled trial[Publication Type]

#2 "random*"[Text Word] OR allocation[Text Word] OR "random allocation"[Text Word] OR placebo[Text Word] OR single blind[Text Word] OR double blind[Text Word] OR "randomized controlled trial*"[Text Word] OR RCT[Text Word]

#3 #1 OR #2

#4 animals NOT humans

#5 #3 NOT #4

#6 chronic disease[Mesh] OR "cardiovascular diseases"[Mesh] OR "heart disease"[Mesh] OR stroke[Mesh] OR or "non-communicable diseases" OR "non-infectious diseases" OR "non-transmissible diseases" OR "heart"[Title/Abstract] or stroke[Title/Abstract]

#7 tai chi[Title/Abstract] OR "taiji*"[Title/Abstract] OR qigong[Title/Abstract] OR liuzijue[Title/Abstract] OR wuqinxi[Title/Abstract] OR yijinjing[Title/Abstract] OR baduanjin[Title/Abstract] OR "traditional exercise"[Title/Abstract] OR traditional chinese medicine[Title/Abstract] OR"chinese traditional exercise" OR " traditional chinese exercise" OR "chinese exercise"

#8 #5 AND #6 AND #7

2. Search Strategy for EMBASE:

#1 'randomization'/exp OR 'placebo'/exp OR 'placebo effect'/exp OR 'single blind procedure'/exp OR 'double blind procedure'/exp OR 'randomized controlled trial'/exp OR 'randomized controlled trial (topic)'/exp OR 'controlled clinical trial'/exp OR 'controlled clinical trial (topic)'/exp OR 'clinical trial'/exp OR 'clinical trial (topic)'/exp

#2 random*:ab,ti OR allocation:ab,ti OR "random allocation":ab,ti OR placebo:ab,ti OR single blind:ab,ti OR double blind:ab,ti OR randomised controlled trial*:ab,ti OR randomized controlled trial*:ab,ti OR RCT:ab,ti OR clinical trial*:ab,ti

#3 #1 OR #2

#4 tai chi:ab,ti OR taiji*:ab,ti OR qigong:ab,ti OR liuzijue:ab,ti OR wuqinxi:ab,ti OR yijinjing:ab,ti OR baduanjin:ab,ti OR traditional exercise:ab,ti OR chinese traditional exercise:ab,ti OR traditional chinese exercise:ab,ti OR chinese exercise:ab,ti

#5 #3 AND #4

3. Search Strategy for Cochrane Library

#1 "random*" or allocation or "random allocation" or placebo or single blind or double blind or "randomized controlled trial*" or RCT or "clinical trial*"

#2 randomized controlled trial:pt or clinical trial:pt

#3 tai chi:ti,ab,kw OR taiji*:ti,ab,kw OR qigong:ti,ab,kw OR liuzijue:ti,ab,kw OR wuqinxi:ti,ab,kw OR yijinjing:ti,ab,kw OR baduanjin:ti,ab,kw OR traditional exercise:ti,ab,kw OR chinese traditional exercise:ti,ab,kw OR traditional chinese exercise:ti,ab,kw OR chinese exercise:ti,ab,kw

#4 #1 and #2 and #3

4. Search Strategy for CINAHL (Ebsco)

S1 MH("Random Assignment" OR "Placebos" OR "Placebo Effect" OR "Single-Blind Studies" OR "Double-Blind Studies" OR "Triple-Blind Studies" OR "Randomized Controlled Trials" OR "comparative studies" OR "Evaluation Research" OR "Prospective Studies" OR "crossover Design" OR "Prospective Studies" OR "Clinical Trials" OR "Clinical Trial Registry")

S2 TX(random$ OR allocation OR "random allocation" OR placebo$ OR single blind OR double blind OR "randomi?ed controlled trial*" OR "controlled clinical trial*" OR "comparative study" OR "evaluation stud*" OR "follow-up stud*" OR "prospective stud*" OR "cross-over stud*" OR control$ OR prospectiv$ OR volunteer$ OR "RCT" OR "clinical trial*")

S3 PT(randomized controlled trial OR "clinical trial*")

S4 S1 OR S2 OR S3

S5 AB(tai chi OR taiji OR qigong OR liuzijue OR wuqinxi OR yijinjing OR baduanjin OR traditional exercise OR chinese traditional exercise OR traditional chinese exercise OR chinese exercise)

S6 S4 AND S5

5. Search Strategy for web of science

#1 TS=("random*" OR allocation OR "random allocation" OR placebo OR single blind OR single blind method OR double blind OR double blind method OR "randomized controlled trial*" OR "randomised controlled trial*" OR "RCT" OR "clinical trial*")

#2 TS=(tai chi OR taiji OR qigong OR liuzijue OR wuqinxi OR yijinjing OR baduanjin OR traditional exercise OR chinese traditional exercise OR

traditional chinese exercise OR chinese exercise)

#3 TS=(chronic disease OR "cardiovascular diseases" OR "heart disease" OR stroke OR "non-communicable diseases" OR "non-infectious diseases" OR "non-transmissible diseases")

#4 #1 AND #2 AND #3

Timespan=All years. Databases=SCI-EXPANDED, SSCI, A&HCI, CPCI-S, CPCI-SSH.

6 Search Strategy for CNKI

#1 SU=随机 OR SU=随机分配 OR SU=随机对照 OR SU=对照 OR SU=盲法 OR SU=单盲 OR SU=双盲 OR SU=随机对照试验 OR SU=随机对照研究 OR SU=临床试验 OR SU=临床观察 OR SU=临床研究（精确匹配）

#2 SU=太极拳 OR SU=气功 OR SU=六字诀 OR SU=易筋经 OR SU=五禽戏 OR SU=八段锦 OR SU=传统训练 OR SU=中国传统运动训练 OR SU=中国训练（精确匹配）

#3 #1 AND #2
